# Novel enzymes for the degradation of cellulose

**DOI:** 10.1186/1754-6834-5-45

**Published:** 2012-07-02

**Authors:** Svein Jarle Horn, Gustav Vaaje-Kolstad, Bjørge Westereng, Vincent GH Eijsink

**Affiliations:** 1 Department of Chemistry, Biotechnology and Food Science, Norwegian University of Life Sciences, P.O. Box 5003, Aas, Norway

**Keywords:** Cellulase, Cellulose, GH61, CBM33, Biofuel, Bioethanol, Lytic polysaccharide monooxygenase, Biorefinery, Bioeconomy, Aldonic acid

## Abstract

The bulk terrestrial biomass resource in a future bio-economy will be lignocellulosic biomass, which is recalcitrant and challenging to process. Enzymatic conversion of polysaccharides in the lignocellulosic biomass will be a key technology in future biorefineries and this technology is currently the subject of intensive research. We describe recent developments in enzyme technology for conversion of cellulose, the most abundant, homogeneous and recalcitrant polysaccharide in lignocellulosic biomass. In particular, we focus on a recently discovered new type of enzymes currently classified as CBM33 and GH61 that catalyze oxidative cleavage of polysaccharides. These enzymes promote the efficiency of classical hydrolytic enzymes (cellulases) by acting on the surfaces of the insoluble substrate, where they introduce chain breaks in the polysaccharide chains, without the need of first “extracting” these chains from their crystalline matrix.

## Introduction

Biomass in the form of bioenergy provides about 10% of the global energy supply (50 EJ/year), and is the largest source of renewable energy. Most current biomass use concerns traditional burning in developing countries for heating and cooking, while biofuels (bioethanol and biodiesel) represent about 3 EJ/year. All harvested biomass currently used for food, fodder and fibre equals approximately 219 EJ/year. A three-fold increase in the use of bioenergy, to 150 EJ/year, would require nearly the entire current global biomass harvest [1]. Nevertheless, it has been estimated that the potential deployment level of biomass for energy by 2050 could be in the range 100 to 300 EJ. Since liquid transportation fuels are less easy to replace than heat and power, future use of biomass for energy is likely to focus on the former.

Modern applications of bioenergy are based on convenient solid, liquid and gaseous energy carriers, typical examples being pellets, bioethanol and methane. The biofuel produced in biggest volume today is bioethanol with an annual production of 84 billion litres (2010) projected to reach 125 billion litres in 2017 [2]. Currently, bioethanol is mainly produced from starch (corn in the US) or sugar (sugarcane in Brazil). However, starch and sugar are also potential food sources and great efforts are being made to develop biofuels based on non-food biomass such as lignocellulosic or algal biomass. These so-called second generation biofuels may be produced through thermochemical processes [3], such as pyrolysis, or through biochemical processes. This paper addresses important recent developments related to biochemical conversion of biomass, in particular the enzymatic conversion of plant polysaccharides to monomeric sugars, the central “platform chemical” of the future biorefinery.

Biochemical conversion of biomass advantageously preserves the original carbohydrate structures in the form of monomeric sugars (in contrast to thermochemical conversion which leads to destruction of the carbohydrates) and enzyme technology is generally considered the most sustainable technology for saccharification. However, despite large efforts in the past decade, the (in)efficiency of enzymatic hydrolysis of lignocellulosic materials remains a key limiting step in many biorefining approaches [4]. Limiting factors lie in the heterogeneity of the plant cell wall (primarily cellulose, hemicelluloses and lignin [5]) and the inaccessibility and recalcitrance of its individual components.

Traditionally, enzyme systems capable of degrading recalcitrant polysaccharides, such as cellulose, are thought to consist of endo-acting enzymes that cut randomly in the polysaccharide chain, and processive exo-acting enzymes that degrade the polymers from chain ends [6]. However, polysaccharide chains in a crystal are tightly packed and the existence of additional factors that would make the substrate more accessible has been suggested since the 1950s [7]. Recent studies of bacterial proteins currently classified as family 33 Carbohydrate Binding Modules (CBM33) [8-11] and of fungal proteins currently classified as family 61 Glycoside Hydrolases [12-18] have shown that the classical endo/exo scheme indeed may be too simple. These proteins have flat substrate-binding surfaces and are capable of cleaving polysaccharide chains in their crystalline contexts using an oxidative mechanism that depends on the presence of divalent metal ions and an electron donor [8]. CBM33- and GH61-encoding genes are abundant in the genomes of biomass-converting microorganisms and these oxidative enzymes represent a new paradigm for degradation of recalcitrant polysaccharides that may be of major importance for the future biorefinery.

## Lignocellulosic biomass and processing

### Lignocellulose

Lignocellulosic plant biomass consists mainly of three types of polymers: lignin, cellulose and hemicellulose. These three polymers are interlinked in a hetero-matrix and their relative abundance varies depending on the type of biomass [19]. Examples of such biomass are angiosperms (hardwoods), gymnosperms (softwoods) and graminaceous plants (grasses such as wheat, giant reed and Miscanthus). The main components of lignocellulosic biomass are cellulose (40–50%), hemicellulose (20–40%) and lignin (20–30%). Minor components are proteins, lipids, pectin, soluble sugars and minerals [20].

**Figure 1:**
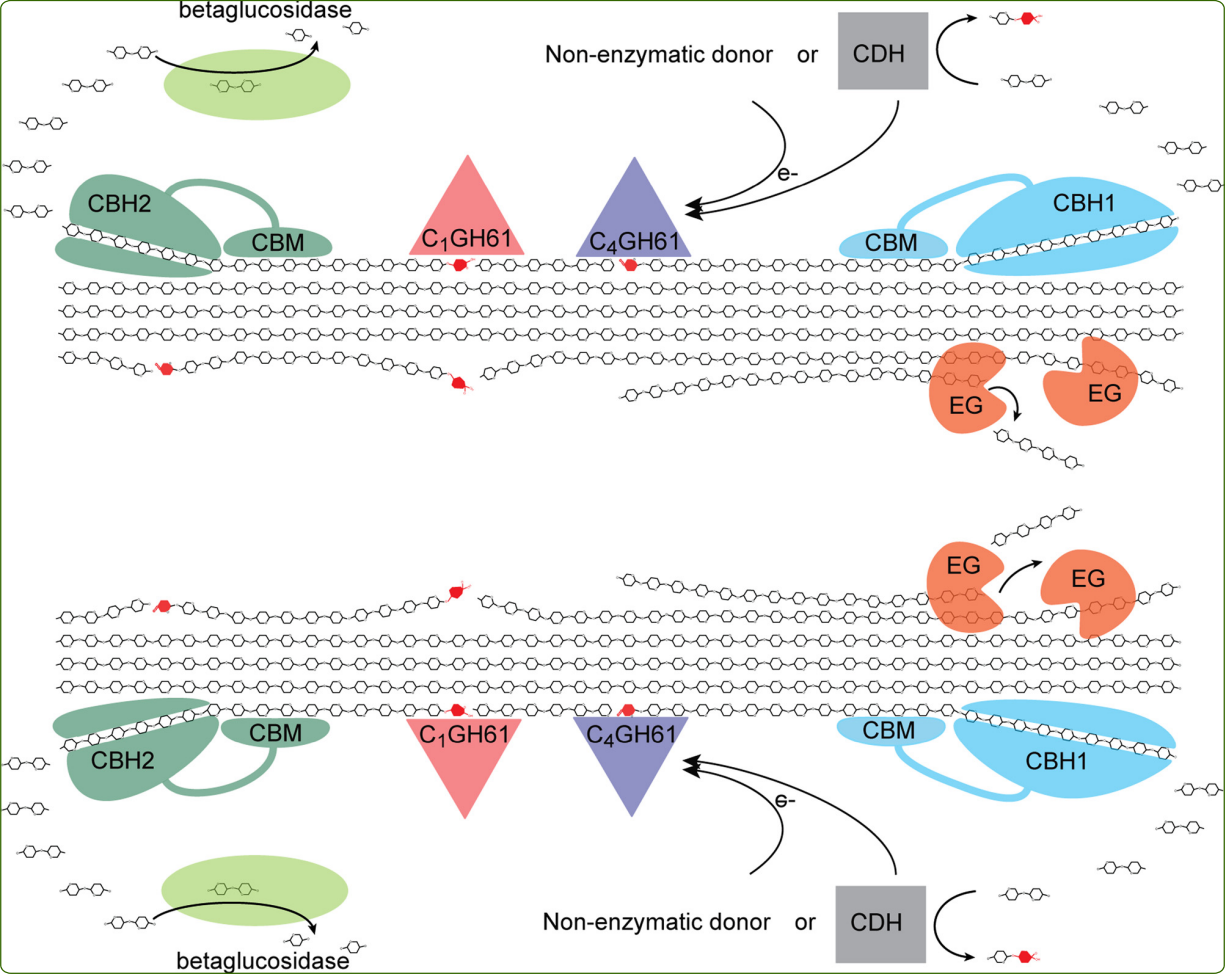
**Structural overview of a cellulose chain (A) and a simplistic sketch of a Iβ cellulose microfibril (B).** Note the simplicity and homogeneity of the cellulose chain. Parallell cellulose chains aggregate into crystalline structures called microfibrils. The arrows indicate the two hydrophobic faces [26] of the microfibril which are thought to be attacking points for cellulases [27].

Cellulose is a linear polysaccharide consisting of hundreds to over ten thousand β-1,4 linked glucose units (Figure 1A). The cellulose chains aggregate into microfibrils via hydrogen bonding and van der Waals interactions [21,22], reported to consist of 24 to 36 chains based on scattering data [23] and information about the cellulose synthase [24], respectively (Figure 1B). These microfibrils are crystalline and non-soluble and enzymatic saccharification is challenging. Consecutive sugars along chains in crystalline cellulose are rotated by 180 degrees, meaning that the disaccharide (cellobiose) is the repeating unit. Cellulose tends to contain both well ordered crystalline regions and disordered, more amorphous regions. In nature crystalline cellulose is found as parallel chains in the form of Iα and Iβ, where Iβ is the predominant form in plants (Figure 1B). Pretreatment (see below) may lead to the formation of other types of crystalline cellulose (i.e. type II, III and IV) [25]. While its recalcitrance to enzymatic degradation may pose problems, one big advantage of cellulose is its homogeneity. Complete depolymerization of cellulose yields just one product, glucose. 

The term hemicellulose collectively names non-cellulose polysaccharides that show large variation, within one plant species and its tissues and in between plants. Common hemicelluloses are xylan, abundant in grasses and angiosperms (hardwoods like birch and aspen), mannan, abundant in gymnosperms (softwoods like spruce and pine), and xyloglucan, abundant in many angiosperms. Hemicelluloses are heteropolymers with varying degrees of branching. This may be exemplified by hardwood xylans which have a β-1,4-linked xylose backbone with a high amount of acetylesterifications and a lesser amount of α-1,2 linked glucuronic acid/4-O-Methyl-glucuronic acid substituents [28]. Grass xylan is more complex, containing a high degree of arabinose substitutions and ester modifications like acetylesterification and hydroxycinnamic acid esters (p-coumaric acid and ferulic acid) [29]. Xylan chains may be cross-linked via hydroxycinnamic acids [30]. Glucomannan contains a mixed β-1,4-linked mannose/glucose backbone substituted with α-1,6-linked galactose and with some mannose residues O-2/O-3 acetylesterified [31].

Hemicelluloses are generally easier to degrade enzymatically than cellulose but certain oligomeric structures are recalcitrant because of complex branching and acetylation patterns [32]. Hemicellulose structures may add to the recalcitrance of cellulose and enzymes such as xylanases are common in industrial enzyme cocktails for lignocellulose processing [33]. While depolymerization of cellulose only yields glucose, degradation of hemicelluloses yields a mixture of different sugars that may contain substantial amounts of pentoses that are difficult to ferment.

Lignin is a relatively hydrophobic and aromatic heteropolymer consisting of three monolignols, methoxylated to various degrees: coniferyl alcohol, sinapyl alcohol and p-coumaryl alcohol. These monolignols are incorporated into lignin in the form of guaiacyl (G), syringyl (S) and p-hydroxyphenyl (H), respectively. The relative amounts of these monolignols vary between different sources of lignin [34]. Softwoods have lignins dominated by G, whereas hardwood lignin is a mix of G and S. Lignin from grasses typically contains all three types of monolignols [34,35]. In lignocellulosic biomass lignin is crosslinked with carbohydrates by ether or ester linkages via e.g. arabinose-ferulic acid or glucuronic acid [36].

Enzymes known to act on lignin are mostly co-factor dependent oxidoreductases [37], which implies that their industrial use is going to be expensive. Furthermore, today, there is no known simple enzymatic scenario for depolymerization of lignin. Interestingly, the ability of microbes to degrade aromatic compounds such as lignin building blocks is well documented [38].

### The biorefinery and the key role of enzymes

The cellulose-hemicellulose-lignin matrix is highly recalcitrant and thus not efficiently degraded to sugars by enzymes alone. Therefore, some kind of pretreatment is usually applied to make the biomass more accessible to enzymes [19]. Chemical methods for polysaccharide depolymerization do exist, but most biorefining strategies pursued world-wide are based on the use of enzymes. Depolymerization of pretreated biomass is achieved by adding an enzyme cocktail which degrades the polysaccharides to pentoses (xylose and arabinose) and hexoses (glucose, mannose and galactose). The most commonly used commercial enzyme cocktails are produced by the fungus * Trichoderma reesei * (nowadays called * Hypocrea jecorina *) and the depolymerization process usually takes place at a pH 4.5 - 5.0 and temperatures in the range of 40 to 50°C.

## Enzymatic degradation of cellulose

### Classical view

The classical scheme for cellulose degradation involves the synergistic action of three classes of enzymes:

1) Endo-1,4-β-glucanases randomly cleave internal bonds in the cellulose chain. These enzymes may be non-processive or processive (in processive enzymes, enzyme-substrate association is followed by several consecutive cuts in a single polysaccharide chain that is threaded through the active site [39-41]).

2) Exo-1,4-β-glucanases attack the reducing or non-reducing end of the cellulose polymer. Processive exo-1,4-β-glucanases are referred to as cellobiohydrolases; they are among the most abundant components in natural and commercial cellulase mixtures and a subject of intense study.

3) β-glucosidases convert cellobiose, the major product of the endo- and exo-glucanase mixture, to glucose.

These enzymes act synergistically because endo-acting enzymes generate new reducing and non-reducing chain ends for the exo-acting enzymes, which release cellobiose that is converted to glucose by β-glucosidases [6,42,43]. It is important to note that natural cellulolytic enzyme systems often contain several exo- and endo-acting enzymes which may have varying preferences for varying forms of cellulose (crystalline versus amorphous; specific crystal faces [6,43-45]). Variation in affinity for the various forms of cellulose may in part be a consequence of variation in the presence of Carbohydrate-Binding Modules (CBMs) that are covalently attached to the catalytic domains of the enzymes in question [44,46,47].

All these enzymes are hydrolases, i.e. they cleave glycosidic bonds by addition of a water molecule [48]. Commercial cellulase mixtures are mainly based on the cellulolytic enzyme cocktail produced by * H. jecorina * which is dominated by processive cellobiohydrolases (up to 80% of the proteins) [49]. Processivity is probably essential to effectively degrade the most crystalline parts of cellulose. It has been pointed out, however, that processive glycoside hydrolases are intrinsically slow [39,50] and that, therefore, well pretreated cellulose, with more amorphous regions, perhaps could be more efficiently degraded with a cellulase mixture containing less processive enzymes [50].

### A new discovery – oxidative cleavage of chitin

As early as 1950, Reese and co-workers suggested that hydrolysis of cellulose would require a non-hydrolytic component that could disrupt polymer packing in the substrate, thereby increasing its accessibility for hydrolytic enzymes [51]. In 2005 it was discovered that a bacterium that breaks down chitin, a crystalline analogue of cellulose occurring in the shells of insects and crustaceans, produces a protein (CBP21) that increases substrate accessibility and potentiates hydrolytic enzymes [52]. This protein was (and, at the time of writing, still is) classified as a family 33 carbohydrate-binding module (CBM33) in the Carbohydrate Active Enzymes (CAZy) database [11]. In a later study, David Wilson and co-workers showed that CBM33 proteins from * Thermobifida fusca * potentiate chitin hydrolysis by chitinases and, possibly, cellulose hydrolysis by cellulases [53].

**Figure 2:**
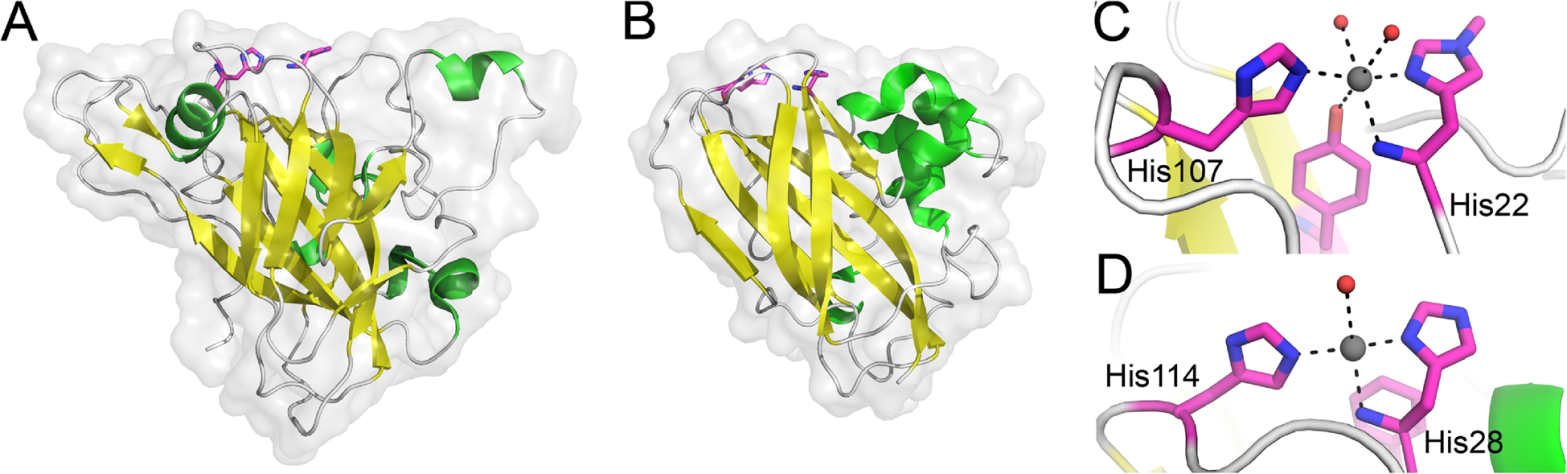
**Structures of CBM33s and GH61s.** The figure shows * Ta *GH61A, a cellulose-active GH61 from * Thermoascus aurantiacus * (**A**), CBP21, a chitin-active CBM33 from * Serratia marcescens * (**B**), and details of the active sites of these two enzymes (**C & D**, respectively). Panels A and B show cartoons of the complete proteins; the side chains of two conserved histidines, which are labeled in panels C & D, are also shown. The grey balls in panels C and D represent metal ions (see text for details); the red balls indicate water molecules. Note that the histidines labeled His22 and His28 in panels C and D, respectively, are the N-terminal residues of the mature proteins (i.e. after removal of the signal peptide for secretion) and that the N-terminal amino group participates in coordination of the metal ion.

Genes coding CBM33s are common in bacteria and viruses but rare in eukaryotes. However, fungi produce proteins currently classified as family 61 glycoside hydrolases (GH61) that are structurally similar to CBM33 proteins [54] (Figure 2) and that act synergistically with cellulases [12]. The structural similarity includes a diagnostic conserved arrangement of the N-terminal amino group and two histidines that may bind a metal ion (Figure 2). One of these histidines (His28/His22 in Figure 2) is the N-terminal residue of the mature secreted protein. 

**Figure 3:**
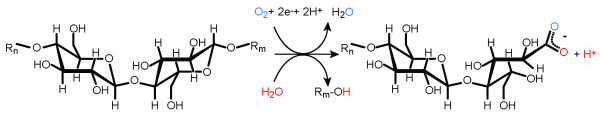
**Summary of the oxidative cleavage of cellulose.** In the case of cleavage by CelS2, a CBM33, and * Pc *GH61D [17] the only oxidized sugars observed are aldonic acids, as indicated in this figure. Other members of the GH61 family seem to generate additional oxidized species, with oxidation at C4 or C6 (see Quinlan et al. [13] and Phillips et al. [16] for further discussion).

Until 2010, it was unclear how CBM33s and GH61s work. Enzymatic activities had not been shown but it was clear that these proteins somehow increased substrate accessibility for hydrolytic enzymes [12,50,52]. However, in a landmark study late in 2010 it was shown that CBP21 is an enzyme which cleaves glycosidic bonds in chitin in an oxidative manner, generating a normal non-reducing chain end and a chain end comprising a C1-oxidized sugar called aldonic acid [8]. It was also shown that the activity of CBP21 is boosted by adding electron donors such as ascorbic acid and that enzyme activity depends on the presence of divalent metal ions and thus may be inhibited by chelators such as EDTA. Isotope labelling confirmed that the reaction involved molecular oxygen, O_2_ (Figure 3). 

### Oxidative cleavage of cellulose

**Figure 4:**
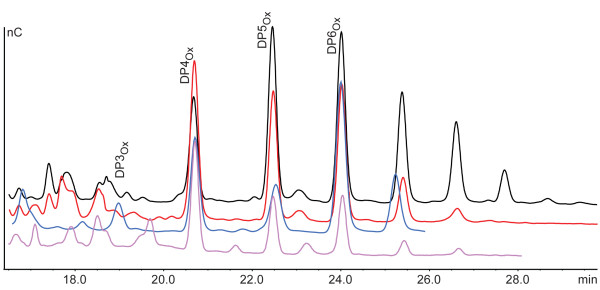
**HPLC analysis of oxidized products generated by CelS2 and PcGH61D.** The main peaks represent aldonic acids of varying chain length (DP, degree of polymerization), as indicated. These soluble products are generated when the same cellulose chain is cut twice by the enzyme, and when the number of sugar units in between the cleavage sites is sufficiently low (longer oligomers are not soluble). The resulting oligomeric products have normal non-reducing ends and are oxidized at the other end. The color coding is as follows: Phosphoric acid swollen cellulose + *Pc*GH61D (black), Avicel + *Pc*GH61D (red), Cellulose nanofibrils + *Pc*GH61D (magenta), and Avicel + CelS2 (blue). Note that the enzymes also produce small amounts of native oligomers [9,17] (not shown in figure). This is most likely the result of a chain being cleaved close to an already existing reducing chain end. It can, however, not be completely excluded that cleavage without oxidation (i.e. normal hydrolysis) occurs under certain conditions. Figure taken from Westereng et al., 2011 [17].

Inspired by the findings for CBP21 [8] and earlier indications that certain CBM33s may act synergistically with cellulases [53] studies were initiated to see if certain CBM33s act on cellulose like CBP21 acts on chitin. In 2011 it was then shown that CelS2, a CBM33 protein from * Streptomyces coelicolor *, indeed cleaves cellulose, producing aldonic acids [9] (Figure 4). Like CBP21, the activity of CelS2 depended on the presence of divalent metal ions as shown by the inhibitory effect of EDTA and the ability to restore activity by adding divalent metal ions. Again like CBP21, purified CelS2 was active without the addition of metals, probably due to high affinity binding. Both the initial study on CBP21 and the study on CelS2 concluded that the enzymes could use several divalent metal ions, but the most recent work clearly shows that these enzymes in fact are copper-dependent monooxygenases (see below). 

At the same time, a series of elegant studies showed that GH61s are functionally very similar to CBM33s [13,14,16-18]. Quinlan and co-workers [13] described the crystal structure of a GH61 from * Thermoascus aurantiacus * (* Ta *GH61A) and showed that this protein catalyzes oxidative cleavage of cellulose in the presence of an external electron donor such as gallic acid. These authors were the first to convincingly show that enzyme activity is copper-dependent. These findings were confirmed by work on a GH61 from * Phanerochaete chrysosporium * (* Pc *GH61D) by Westereng et al. [17], and work on several GH61 proteins from * Neurospora crassa *[14,16,18]. Thus, GH61s are copper dependent lytic polysaccharide monooxygenases. Recent work on a chitin-active CBM33, using experimental conditions that ruled out possible effects of metal ions trapped in the substrate, showed that this CBM33 was copper-dependent too [10].

Interestingly, the work on * Ta *GH61A and the * N. crassa * GH61 proteins showed that these enzymes not only oxidize the C1 carbon, but also may oxidize C4 or perhaps even C6 [13,14,16]. In our own studies of various CBM33s and * Pc *GH61D we have only observed C1 oxidation (Figure 4), but the studies on * Ta *GH61A and the * N. crassa * GH61s show other products too [13,14,16]. A very recent study convincingly showed that * N. crassa * contains C1 and C4 oxidizing GH61s [14], whereas possible C6 oxidation has been suggested for * Ta *GH61A [13]. It is also plausible that some enzymes will be less specific and may oxidise either the C1 or the C4 position during polysaccharide cleavage. There are conspicuous differences among GH61 sequences that in principle could explain functional differences [17,18]. It should be noted that the position of the oxidation may have implications for synergy with cellulases. Cellobiohydrolases that attack the non-reducing end of the cellulose chain would probably benefit from this end not being modified, as in the case of C1 oxidation (Figure 3). For cellobiohydrolases that attack the reducing end of the cellulose chain, C4 oxidation combined with generation of normal reducing ends could be more favourable.

It should be noted that cellulose degradation by blends of cellulases and oxidative enzymes will produce monomeric and dimeric oxidised sugars (gluconic- and cellobionic acid in the case of C1 oxidation [55]) and that this may affect important aspects of the degradation process, such as product inhibition. Interestingly, cellobionic acid is known to be less inhibitory for cellulases than cellobiose [56]. On the other hand though, cellobionic acid is less readily hydrolyzed by β-glucosidases and the resulting gluconic acid shows stronger product inhibition than glucose [55]. The inhibitory effect of C4 oxidised sugars (4-ketoaldoses) is not known. As for further processing, it has been shown that gluconic acid can be fermented to ethanol [57].

The catalytic mechanism of CBM33s and GH61s remains a subject of intense research and speculations about the mechanism are beyond the scope of this Paper (see [14] and [18] for interesting discussions). For practical purposes, the enzymes’ dependence on copper, molecular oxygen and an external electron donor is of major importance. As to the latter, current data indicate that many different reducing agents can do the job, including ascorbic acid, gallic acid and reduced glutathione. Interestingly, some reports indicate that in nature GH61s may receive electrons from the action of cellobiose dehydrogenase [15,16,58], an enzyme that is secreted in concert with GH61 upon cellulose degradation in some fungi [16] and that previously has been thought to provide electrons for “Fenton chemistry”-based biomass depolymerisation [59,60]. In the case of degradation of lignocellulosic substrates GH61 and CBM33 may get electrons from lignin, as it is shown that lignin can take part in redox cycles [61].

### Diversity of GH61 and CBM33 proteins

Genes encoding GH61 or CBM33 proteins are classified and listed in several gene annotation/classification databases available on the world wide web. The most used databases for carbohydrate active enzymes are CAZy [62] and Pfam [63]. The CAzY database is dedicated to carbohydrate active enzymes and is the only of these databases that is 100% manually curated (meaning good quality of the data). GH61s, i.e. members of the Glycoside Hydrolase family 61, are almost exclusively found in fungi (two annotations in the maize genome form the only exception) and are often abundant in wood degrading fungi. According to Pfam, the genome of the soil fungus * Chaetomium globosum * contains up to 44 unique GH61 encoding genes, of which at least 33 seem to encode complete GH61 modules that are very diverse in sequence. Sequence diversity is generally high in the GH61 family, which could indicate adaptation to other substrates than cellulose, the only substrate known so far.

Many families of carbohydrate-active enzymes are modular, with catalytic domains being linked to additional CBMs (carbohydrate-binding domains) that may endorse enzymes within one family with varying substrate affinities. Despite their large sequence variability, the GH61s show little variation in terms of their modular nature: of the 534 sequences annotated as GH61 in the Pfam database (Pfam ID: PF03443), 409 have no additional modules, whereas 100 have one additional CBM1 module (CBM1 modules are known to predominantly bind cellulose [45,47]). The few remaining GH61s have additional modules with mostly obscure and uncertain annotations. Although it is conceivable that action on crystalline cellulose may require several GH61s that attack various faces on the crystals [18] the massive abundance of GH61-encoding genes in biomass converting fungi does suggest that activity on other polysaccharide substrates may occur. In fact induction of GH61 production by non-cellulose polysaccharides has been observed [64]. Cellulose-binding CBMs may have proximity effects [65] that facilitate GH61 action on other polysaccharides in the lignocellulosic matrix. Interestingly, the most common production strain for commercial cellulase enzyme cocktails (* H. jecorina *) expresses only two GH61 proteins.

**Figure 5:**
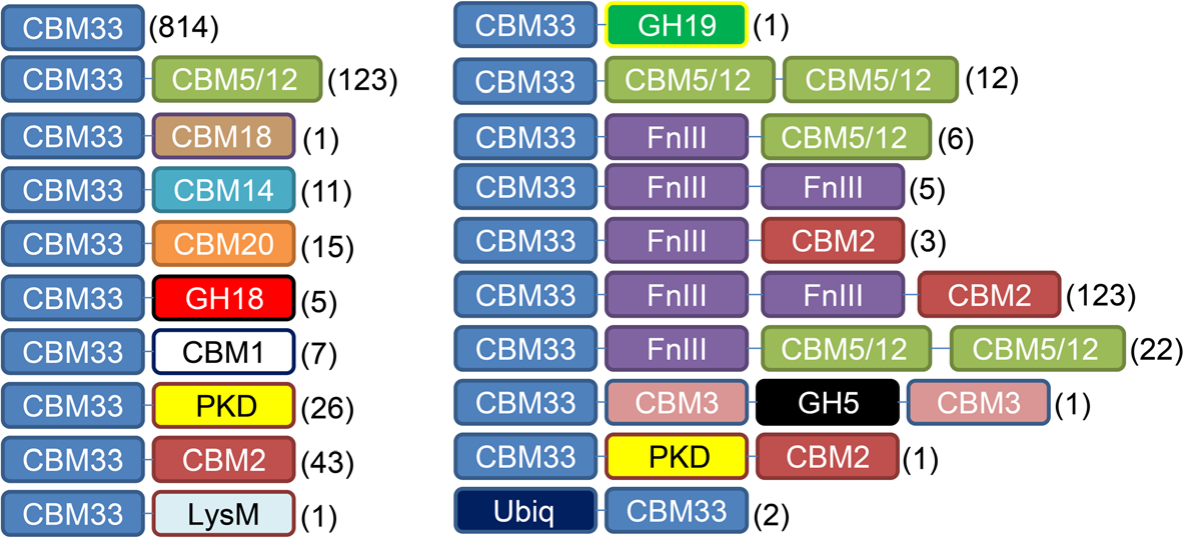
**Domain structure of naturally occurring CBM33-containing proteins.** Annotations are based on Pfam (http://pfam.sanger.ac.uk) and the number of sequences currently representing each architecture is indicated in brackets. All module families shown are themselves diverse, but have been show experimentally to have (at least) the following substrate preferences: CBM33, chitin, chitosan, cellulose; CBM1, cellulose and chitin; CBM2, chitin, cellulose and xylan; CBM5/2, chitin and cellulose, FnIII, a wide variety of soluble and insoluble substrates; CBM20, granular starch and cyclodextrins; CBM18, chitin; CBM3, cellulose and chitin; CBM14, chitin; PKD (Polycystic kidney disease protein like protein), unknown substrate; LysM, peptidoglycan. Three hydrolytic modules are also present: GH5 (cellulose/mannan/chitosan/xylan and more), GH18 (chitin, chitosan) and GH19 (chitin, chitosan). Note that the CBM33 module almost exclusively occurs at the N-terminus, in accordance with the notion that the N-terminal histidine is crucial for activity (Figure 2).

High sequence diversity is also observed in the CBM33 family. However, CBM33s are not limited to a specific kingdom but are represented in a variety of organisms, including viruses, bacteria, fungi and insects (CBM33s are rarely found in anaerobic microbial communities, including bacteria known to produce cellulosomes). Approximately 30% of the genes annotated as CBM33s (Pfam; ID PF03067) also contain one or several additional binding modules that may increase and/or dictate substrate specificity or other functions (Figure 5). In seven (out of 1222) cases, the CBM33 is fused to a catalytic domain (five GH18, one GH19 and one GH5). CelS2, the first CBM33 protein shown to catalyze oxidative cleavage of crystalline cellulose [9], has a cellulose binding module (CBM2) that binds strongly to the substrate (Forsberg & Vaaje-Kolstad, unpublished observations). Interestingly, the carbohydrate binding modules associated with proteins in the CBM33 family range in substrate specificities from insoluble crystalline substrates (e.g. cellulose and chitin) to polysaccharides of a less crystalline and more soluble nature (e.g. xylan or mannan), indicating that the polysaccharides targeted by this enzyme family may be very diverse. In addition to the observed sequence and modular diversity, their wide-spread occurrence also indicates that CBM33s may have a wide variety of functionalities. So far, activity on cellulose and chitin has been convincingly demonstrated, but there are indications that CBM33s are involved in processes other than cellulose or chitin turnover. Some studies show that CBM33s may be involved in adhesion of bacteria to glycoproteins in the gut (both pathogens and commensals [66,67]), whereas other studies indicate their involvement in substrate recognition [68]. A CBM33 single domain protein is strongly up-regulated as part of the stress response of * Enterococcus faecalis *[69]. There is also convincing evidence showing CBM33s to be essential for the infectivity of insect-larvae targeting viruses [70,71], which could be a consequence of CBM33 effects on insect chitin. It is conceivable that in some cases the CBM33s have a mere binding function. However, sequence alignments show that the residues currently identified as being important for catalysis [72] are mostly conserved within the family. 

### A new paradigm for cellulose conversion

While the roles and synergistic actions of classical endoglucanases and cellobiohydrolases in the degradation of cellulose are rather well understood [6], several questions and challenges remain. It is possible to degrade celluloses quite effectively with a combination of endoglucanases and cellobiohydrolases, as illustrated by studies with defined enzyme cocktails [49,73]. However, conversion yields are normally well below 100% and achieving high yields when converting a heterogeneous biomass rich in crystalline cellulose requires harsh pretreatments. Furthermore, any improvement in hydrolysis speed, e.g. by increasing cellulose accessibility [74] or changing to a less stable crystalline form [73,75] is obviously of industrial interest. More theoretically, it is difficult to conceive how enzymes would be able to act on densely packed crystalline forms of cellulose. Indeed, molecular dynamics simulations have indicated that considerable work is needed to achieve the degree of decrystallization that would be needed for enzymes to gain access to single cellulose chains [26,76]. Reese et al. anticipated this when hypothesizing about a missing factor in 1950 [51].

**Figure 6:**
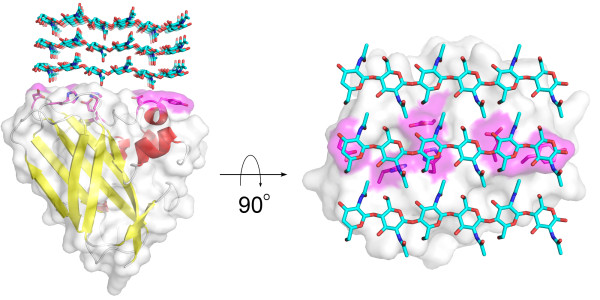
**Artist impression of the interaction between CBP21 and chitin (side view, left; top view, right).** The picture highlights how the flat surface of CBP21 fits the flat surface of a β-chitin crystal (the binding interaction is hypothetical and has not been modelled). The surfaces of residues known to interact with chitin [72] are coloured magenta and the side chains of these residues are shown. In the side view some of the magenta surface is hidden by the white surface of other residues. Please note that the actual orientation of the enzyme relative to the substrate is unknown; see [18] for an interesting discussion of this topic.

**Figure 7:**
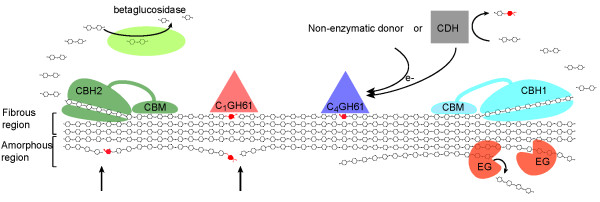
**Current view on fungal enzymatic degradation of cellulose.** Abbreviations: EG, endoglucanase; CBH, cellobiohydrolase; CDH, cellobiose-dehydrogenase; CBM, carbohydrate-binding module. Note that many cellulolytic enzyme systems have multiple EG and/or CBH that may act on various parts of the substrate, e.g. different crystal faces or parts differing in terms of crystallinity and accessibility. The picture shows a C1 and a C4 oxidizing GH61 which would generate optimal (i.e. non-oxidized) ends for the CBH2 and the CBH1, respectively (oxidized sugars are colored red). Note that the combined action of C1 and C4 oxidizing enzymes may produce native cello-oligosaccharides from the middle of the cellulose chain. The possible consequence of GH61 action is illustrated in the lower left part of the picture, where new attacking points for CBHs are indicated by arrows. CDH may provide GH61s with electrons, but it must be noted that not all organisms have genes encoding for both of these enzyme families in their genome (e.g. * Postia placenta * has four genes encoding GH61s, but none encoding CDH [81]). Also other non-enzymatic reductants (electron donors) have been demonstrated to induce oxidative activity (e.g. reduced glutathione, ascorbic acid and gallic acid). For more information on the various glucanases and the mechanisms for their synergy, the reader is referred to Kostylev and Wilson, 2012 [43].

The discovery of oxidative cleavage of cellulose by copper monooxygenases sheds new light on these issues. Perhaps one of the most appealing aspects of these novel enzymes lies in their flat substrate binding sites (Figure 6), which seem well fit to attach to flat crystalline surfaces on the substrate, where they might disrupt packing and generate accessibility, not only by introducing a cut in the polymer but also by the introduction of a charged group (Figure 3). The GH61 and CBM33 enzymes add a completely novel tool to nature’s toolbox of biomass degrading enzymes. The potential importance of this tool is supported by recent transcriptomics and proteomics studies showing that the expression of some GH61s and cellulose-active CBM33s is induced by cellulose and/or co-regulated with the expression of cellulases [77-80]. All in all, these recent findings suggest a new paradigm for the enzymatic degradation of cellulose where the action of classical hydrolytic cellulases is facilitated by the action of these lytic polysaccharide monooxygenases, as illustrated in Figure 7. 

More work is needed to further refine the model depicted in Figure 7 and to determine how general this model is. Cellulases and their substrate-binding CBMs show great variation in terms of their ability to attack various forms of cellulose [47] and similar variation may occur among members of the GH61 and CBM33 families. Notably, various types of cellulose, e.g. resulting from various types of pretreatment [73] have different accessibilities and show different susceptibilities for cellulases. Thus, the impact of adding surface-active GH61 and CBM33 enzymes to cellulase cocktails is likely to vary depending on the substrate.

So far, only very few CBM33 and GH61 proteins have been successfully overexpressed and characterized. Moreover, characterization work has often been limited to testing only a few reaction conditions (in terms of the type of substrate, the pH of the reaction, the reaction temperature, the type of reductant present, and so on). Kinetic data for cellulose are not yet available, whereas preliminary data for a CBM33 acting on β-chitin indicate that this enzyme is one – two orders of magnitude slower than chitinases acting on the same substrate (i.e. rates in the order of 0.01 – 0.1 s-1; [8]). As to the degree of oxidation, available data for both chitin and cellulose indicate that as many as 5% of the sugars may end up oxidized [8,55]. To get a better insight into the functionality of these enzyme families, more members need to be expressed and these need to be characterized in more detail. Different combinations of these enzymes and a wide selection of hydrolytic cellulases need to be tested on both amorphous and different forms of crystalline cellulose substrates. It would also be of major interest to see how these novel enzymes interact with more natural substrates, for example cellulose-rich plant cell material that has not undergone any form of pretreatment.

## Conclusions and future perspectives

The discovery of lytic polysaccharide monooxygenases currently classified as CBM33 and GH61 may represent a revolution in enzymatic biomass processing, although further work is needed to establish their full potential. From a scientific point of view these enzymes are interesting because they represent a novel type of enzymatic activity. From an applied point of view these enzymes are of interest because they may speed up enzymatic conversion of biomass, thus reducing enzyme loads and processing times. Reported effects on chitinase activity are huge [8,10], whereas reported effects on cellulose activity are less [9,12,17,55] but still significant and of considerable commercial value. One of the best known commercial cellulose preparations today, Cellic CTec2 produced by Novozymes, contains extra GH61s that contribute to this product’s improved performance compared to its predecessors [55]. Although industrial knowledge on these enzymes remains mostly invisible to the outside world, it would seem that accumulated research on CBM33s and GH61s still is quite limited. It is thus likely that further improvements in enzymatic biomass conversion through use of these enzymes will emerge in the coming years.

The classifications of CBM33s and GH61s as carbohydrate-binding modules and glycoside hydrolases, respectively, are clearly wrong and will thus change in the near future. Although the two enzyme families show strong similarities, it remains to be seen how similar they really are. Mechanistically, there may be differences, as indicated by the production of C4 and perhaps even C6-oxidized sugars by some of the GH61s described so far. Also, all GH61 structures published so far show methylation of the N-terminal histidine in the active site [13,18]. Such methylation has not been observed in CBM33s and, to the best of our knowledge, not in bacteria in general [13,18,72,82].

Another issue concerns possible variation in substrate specificity. The multiplicity of genes, especially in the case of GH61s, the large sequence variation (e.g. see [18]) and, in the case of CBM33s, the huge variation in modular structure, all suggest that different substrates are targeted. In addition to the various forms and crystal faces of chitin and cellulose, other complex and ordered structures may be targeted such as junction zones or other carbohydrate aggregates in tightly inter-linked hemicellulose-cellulose chains [83-85]. The physiological data available for some CBM33s certainly support the idea of a wider substrate range within this family.

It is important to note that so far, there are no indications that these novel lytic polysaccharide monooxygenases act on single chains, which in an experimental context would mean soluble oligosaccharides. In this sense these novel enzymes differ dramatically from classical glucanases, which need to position single chains in their active sites grooves, clefts or tunnels [48], and which normally are active on soluble oligosaccharides. Such glucanases may have additional CBMs providing affinity for crystalline surfaces [86]. Since CBM33s and GH61s have extended substrate-binding surfaces, one may wonder how extended and ordered the substrate surfaces need to be. It is conceivable that other plant polysaccharides as well as perhaps even the more complex of the glycans found in glycoproteins, contain enough “surface” (i.e. expanding beyond a single chain), to interact with certain CBM33s and/or GH61s. All in all, we consider it likely that CBM33s and GH61s acting on biomass structures such as xylan, mannan and starch will be discovered in the near future.

While the recent finding of lytic polysaccharide monooxygenases represents a major advance in the development of better enzyme technology for biomass conversion, more is likely to come. Proteins such as “swollenins” [87,88] and expansins [89] have for long been known to affect cellulose and other plant polysaccharides but their use in biomass processing has not yet been fully explored. Certain CBMs may have roles beyond mere substrate-binding and they might have some sort of substrate-disrupting effect, thus increasing accessibility to glucanases [74]. Careful engineering of CBMs and appending them to certain glucanases may yield possibilities that so far have remained under-explored. Finally, current massive studies on the microbiomes of herbivores reveal a plethora of potentially relevant biomass-converting enzymes that need further attention and that seem to include cellulolytic enzyme systems unlike the systems known so far [90-92].

Production of biofuels via enzymatic depolymerization of non-food plant polysaccharides currently receives massive attention and the first commercial production facilities are being built [93]. One major reason for current progress is the drastic reduction in enzyme costs that commercial producers have achieved over the past decade. More improvement is needed though, since enzyme costs remain high and, for some tougher substrates, prohibitive [4]. The developments described above open new avenues for further development of enzyme technology in the field. Interestingly, the novel CBM33 and GH61 enzymes do something very different to their substrates than well known hydrolytic enzymes and their implementation may thus require novel thinking. For example, while glucanases need thermochemically pretreated substrates with disrupted crystallinity and sufficiently accessible single polymer chains, these novel enzymes may handle more compact and inaccessible materials. Thus, further implementation of the possibilities offered by CBM33s and GH61s may not only affect the costs of the enzymatic saccharification step but may also direct further optimization of the preceding pretreatment step and process design in general.

## Competing interests

The authors declare no competing interests.

## Authors’ contributions

SJH and VGHE conceptualised the manuscript and wrote parts of it. GVK and BW wrote parts of the manuscript. BW made most of the Figures. All authors contributed to the literature review. All authors read and approved the final manuscript.
